# Associations of Heart Rate Trajectories with Mortality and AKI Occurrence in Septic Patients: A Retrospective Study from MIMIC-IV and eICU Databases

**DOI:** 10.3390/jcm15176660

**Published:** 2026-08-28

**Authors:** Yang Yang, Hangxiang Du, Zhenliang Wen, Jianguo Tang, Nuolin Liu, Changwei Wei, Sheng Zhang

**Affiliations:** 1 Beijing Key Laboratory of Precision Translational Medicine in Anesthesiology and Pain, Anesthesia and Operation Center, Beijing Chao-Yang Hospital, Capital Medical University, Beijing 100020, China; 2 Department of Critical Care Medicine, Ruijin Hospital, Shanghai Jiao Tong University School of Medicine, No.197 Ruijin 2nd Road, Shanghai 200025, China; 3 Department of Trauma-Emergency and Critical Care Medicine Center (TECCMC), Shanghai Fifth People’s Hospital, Fudan University, Shanghai 200240, China

**Keywords:** group-based trajectory model, heart rate trajectory, acute kidney injury, sepsis

## Abstract

**Background**: Abnormal heart rate is a common early manifestation of sepsis, but the association of the dynamic changes with clinical outcomes remains unclear. This study aimed to investigate the relationship between dynamic heart rate trajectories and 28-day mortality as well as acute kidney injury (AKI) in patients with sepsis. **Methods**: This retrospective cohort study was based on two independent cohorts from the eICU-CRD and MIMIC-IV databases, enrolling 7537 patients with sepsis. Group-based trajectory modeling (GBTM) was used to identify dynamic heart rate trajectories within the first 7 days after ICU admission. Landmark analysis with a landmark time of day 7 to account for immortal time bias. Cox regression and logistic regression analyses were applied to evaluate the associations of different trajectories with 28-day mortality and new-onset AKI risk after the 7-day landmark. Sensitivity analyses confirmed the consistency with the primary analysis. **Results**: Five distinct heart rate trajectories were identified: persistently low-stable (Traj-1), moderate-level gradually increasing (Traj-2), high-baseline rapidly declining (Traj-3), low-level U-shaped (Traj-4), and persistently high (Traj-5). Using Traj-1 as the reference, among patients observed at day 7, patients with Traj-5 had elevated risks of 28-day mortality (all *p* < 0.05) and new-onset AKI after the 7-day landmark (all *p* < 0.05). **Conclusions**: Persistently high heart rate trajectories (Traj-5) were associated with higher risks of 28-day mortality and new-onset AKI among patients observed at day 7. These findings support careful heart rate monitoring in septic patients can provide valuable information into the prognostic association and risk stratification, while residual confounding precludes causal interpretation.

## 1. Introduction

Sepsis is defined as life threatening organ dysfunction caused by a dysregulated host response to infection, representing a major global public health burden with high morbidity, mortality, and substantial consumption of medical resources [1,2]. Despite advances in critical care management, sepsis remains one of the leading causes of mortality in intensive care units (ICUs) worldwide [3,4]. Hemodynamic instability constitutes the core pathophysiological feature of sepsis, and sepsis-induced cardiomyopathy (SICM) further contributes to circulatory deterioration and impaired tissue perfusion, with abnormal heart rate serving as one of the earliest and most prevalent clinical manifestations [5,6]. Tachycardia, heart rate fluctuation, and sustained elevated heart rate reflect sympathetic overactivation, compromised tissue perfusion, excessive systemic inflammation, and autonomic nervous system imbalance, all of which are closely associated with the development of multiple organ dysfunction, particularly acute kidney injury (AKI) [7].

The management of sepsis consists of three core components: control of the primary infection, hemodynamic stabilization, and modulation of the host response [8]. Hemodynamically guided resuscitation provides objective indicators for assessing sepsis severity and monitoring therapeutic responses [9,10]. Heart rate is routinely and continuously monitored in clinical practice, and accumulating evidence indicates that heart rate is not merely a compensatory physiological parameter but also an independent prognostic predictor of adverse outcomes in septic patients [11]. A large-sample study based on a 24 h observation window demonstrated that the time-weighted average heart rate above 85 beats per minute was significantly associated with elevated 28-day mortality in a nonlinear pattern [12]. Recent studies have shown that maximum heart rate was independently linked to 28-day mortality in a U-shaped pattern in septic patients. Both excessively high and low MHR increased death risk [13]. However, most existing studies focus on static heart rate values or short-term fluctuations, overlooking the dynamic characteristics of heart rate as the disease progresses. Focusing on the continuous dynamic trajectories of heart rate in the early stages of the disease can effectively identify patient heterogeneity and the dynamic progression of the disease.

Therefore, based on two large critical care databases (MIMIC IV and eICU-CRD), the present study adopted group-based trajectory modeling (GBTM) to identify distinct heart rate trajectories within the first 7 days of ICU admission among septic patients and explored the association between the different heart rate trajectories over the first 7 days after ICU admission and 28-day mortality and new-onset AKI in septic patients. The study aims to provide the theoretical evidence for identifying high-risk populations and improving treatment protocols for ICU patients.

## 2. Materials and Methods

### 2.1. Study Design and Setting

This retrospective observational study utilized data from two large, publicly available intensive care unit (ICU) databases: the Medical Information Mart for Intensive Care IV (MIMIC-IV) database and the eICU Collaborative Research Database (eICU-CRD) [14,15]. The MIMIC-IV database (version 3.1) contains detailed clinical data on ICU patients treated at Beth Israel Deaconess Medical Center in Boston, MA, USA, between 2008 and 2022. The eICU-CRD database, which includes standardized clinical data of ICU patients from multiple hospitals across the United States between 2014 and 2015. Due to the anonymized nature of the data, the requirement for informed consent was waived. The MIMIC IV and eICU-CRD datasets have received ethical approval from the Institutional Review Board of the Massachusetts Institute of Technology. Access to both databases was granted by completing the data use agreement course and test (certification number: 42442549).

### 2.2. Study Population

Patients were included if they met the following criteria: (1) aged ≥18 years; (2) diagnosed with sepsis according to the Sepsis-3 international consensus definition, defined as suspected or confirmed infection plus an acute increase of ≥2 points in the Sequential Organ Failure Assessment (SOFA) score from baseline; the operational definition of Sepsis-3 in this study follows the widely adopted methodology for MIMIC-IV and eICU-CRD cohorts, including antibiotic administration within 24 h, culture sampling ±72 h, ICD-9/10 codes for infection, and 24 h worst-value SOFA scores [16]; and (3) had the heart rate measurements recorded during the first 7 days after ICU admission. Patients were excluded if they met any of the following conditions: (1) age ≤ 18 years; (2) length of ICU stay < 24 h; (3) without heart rate measurements recorded during the first 7 days; (4) non-index or multiple ICU admissions during the same hospitalization.

### 2.3. Data Collection

The mean of all valid heart rate readings within each 24 h period was used as the daily heart rate. Heart rate was continuously monitored and recorded hourly during ICU admission. Momentary heart rate fluctuations caused by bedside interventions, pain, and agitation are non-sustained noise. Daily averages attenuate these transient disturbances. Based on previous studies and clinical experience, covariates that may affect the prognosis of patients with sepsis were collected and extracted using Structured Query Language (SQL) and adjusted for in the analysis: (1) demographic characteristics: age, sex; underlying comorbidities: hypertension, diabetes mellitus, coronary heart disease, chronic kidney disease, and chronic obstructive pulmonary disease; (3) clinical parameters at ICU admission: SOFA score, body temperature, mean arterial pressure, blood lactate, serum creatinine, and white blood cell count, etc.

### 2.4. Study Outcomes

The primary outcome was defined as 28-day mortality after the 7-day landmark. The secondary outcome was the incidence of new-onset AKI specifically identified as new-onset AKI occurring after day 7 of ICU admission through hospital discharge. According to the Kidney Disease Improving Global Outcomes (KDIGO) criteria, the AKI was defined based on the following: (1) serum creatinine (SCr) elevation ≥ 0.3 mg/dL within 48 h; (2) an increase in SCr to ≥ 1.5 times from baseline, known or presumed to have occurred within the prior 7 days; and (3) urine output < 0.5 mL/(kg·h) for a minimum of 6 h [17]. The baseline for serum creatinine was the first measurement taken at ICU admission.

### 2.5. Statistical Analysis

Continuous variables that conformed to Gaussian distribution were expressed as mean ± standard deviation, and Student’s *t*-test or one-way ANOVA was used for intergroup comparison; those that did not conform to Gaussian distribution were expressed as medians and interquartile ranges (IQRs), and nonparametric tests were used for intergroup comparison. Categorical variables were expressed as counts (percentages), and the χ^2^ test or Fisher’s exact test was used for inter-group comparison.

For all variables in the study, missing value assessment was conducted first; variables with a missing data rate > 20% were excluded from subsequent statistical analyses. For variables with a missing data rate < 20%, five imputed datasets were generated through Multiple Imputation by Chained Equations (MICE) using the “mice” package in R software to minimize the impact of missing values on the results. The percentage of missing data is provided in Table S1. All subsequent association analyses, threshold effect analyses, and subgroup analyses were performed based on these five imputed datasets to ensure the reliability and stability of the statistical results.

Group-based trajectory modeling (GBTM) is a type of mixture modeling, which aims to identify patient subgroups with similar dynamic heart rate trajectories in the overall population. In this study, GBTM was applied to identify distinct heart rate trajectory subgroups within the first 7 days after ICU admission among patients with sepsis and was conducted independently in the eICU-CRD and MIMIC-IV cohorts. Trajectory shapes, polynomial orders, and average posterior probability were separately re-estimated for each database. The optimal trajectory model was determined based on the Akaike Information Criterion (AIC), Bayesian Information Criterion (BIC), average posterior probability of assignment (AvePP ≥ 0.7), and odds of correct classification (OCC > 5). Lower absolute values of AIC and BIC indicate better model fitting [18].

Kaplan–Meier survival curves were plotted to depict the 28-day survival trends among patients stratified by different heart rate trajectory subgroups. Landmark analyses were implemented in R using the “jskm” package. Multivariable Cox proportional hazards regression models were used to analyze the association between distinct heart rate trajectories and 28-day mortality after the 7-day landmark. All patients who died or were discharged within the first 7 days of ICU admission were excluded prior to Cox regression analysis. In the MIMIC-IV cohort, of the initial 4319 septic patients, 135 (deaths or discharges) were excluded prior to the 7-day landmark. In the eICU-CRD cohort, of the initial 3218 septic patients, 56 (deaths or discharges) were excluded prior to the 7-day landmark. Four Cox proportional hazards regression models with incrementally adjusted covariates were constructed. Adjusted Model 1 was adjusted for demographic baseline indicators, including age, body mass index (BMI), and gender. Adjusted Model 2 further incorporated adjustments for chronic comorbidities on the basis of Model 1, including cardiac insufficiency, chronic hepatic insufficiency, chronic renal insufficiency, diabetes mellitus, chronic pulmonary disease, malignant cancer, organ transplantation history, and hypertension. Adjusted Model 3 further incorporated for SOFA, lactate, mean arterial pressure, pacing treatment, mechanical ventilation, fever, bloodstream infection, severe arrhythmias, use of beta-blockers, sedatives, analgesics, antipyretics, vasoactive medications, and atrial fibrillation. Meanwhile, we additionally excluded individuals with AKI, RRT, or ESRD within the 7-day landmark. In the MIMIC-IV cohort, 467 out of the 4184 landmark-survived patients developed incident AKI, RRT, or ESRD; in the eICU-CRD cohort, 220 AKI, RRT, or ESRD events were observed among the remaining 3162 patients, and multivariable logistic regression models with the same hierarchical adjustment strategy were established to explore the association between different heart rate trajectories and the risk of new-onset AKI after the 7-day landmark. Restricted cubic spline (RCS) models were applied to visualize the relationships between heart rate metrics (minimum, maximum, and mean values) and 28-day mortality. Subgroup analyses were performed to evaluate the stability of the association between heart rate trajectories and clinical outcomes, and the study population was stratified according to demographic characteristics and baseline comorbidities. Sensitivity analyses were conducted after excluding patients with severe arrhythmias, use of beta-blockers, pacing treatment and atrial fibrillation sequentially. Additionally, the day-7 landmark multivariable Cox analysis was conducted, which incorporated the same variables that were included in the above-mentioned logistic model, to account for the unequal post-landmark follow-up. Within this Cox analysis, all cases were censored at hospital discharge, death, or day 28, whichever occurred first.

All statistical analyses in this study were performed using R software (version 4.2.2), and *p*-value less than 0.05 was considered statistically significant.

## 3. Results

### 3.1. Identification and Characterization of Heart Rate Trajectories

**Figure 1:**
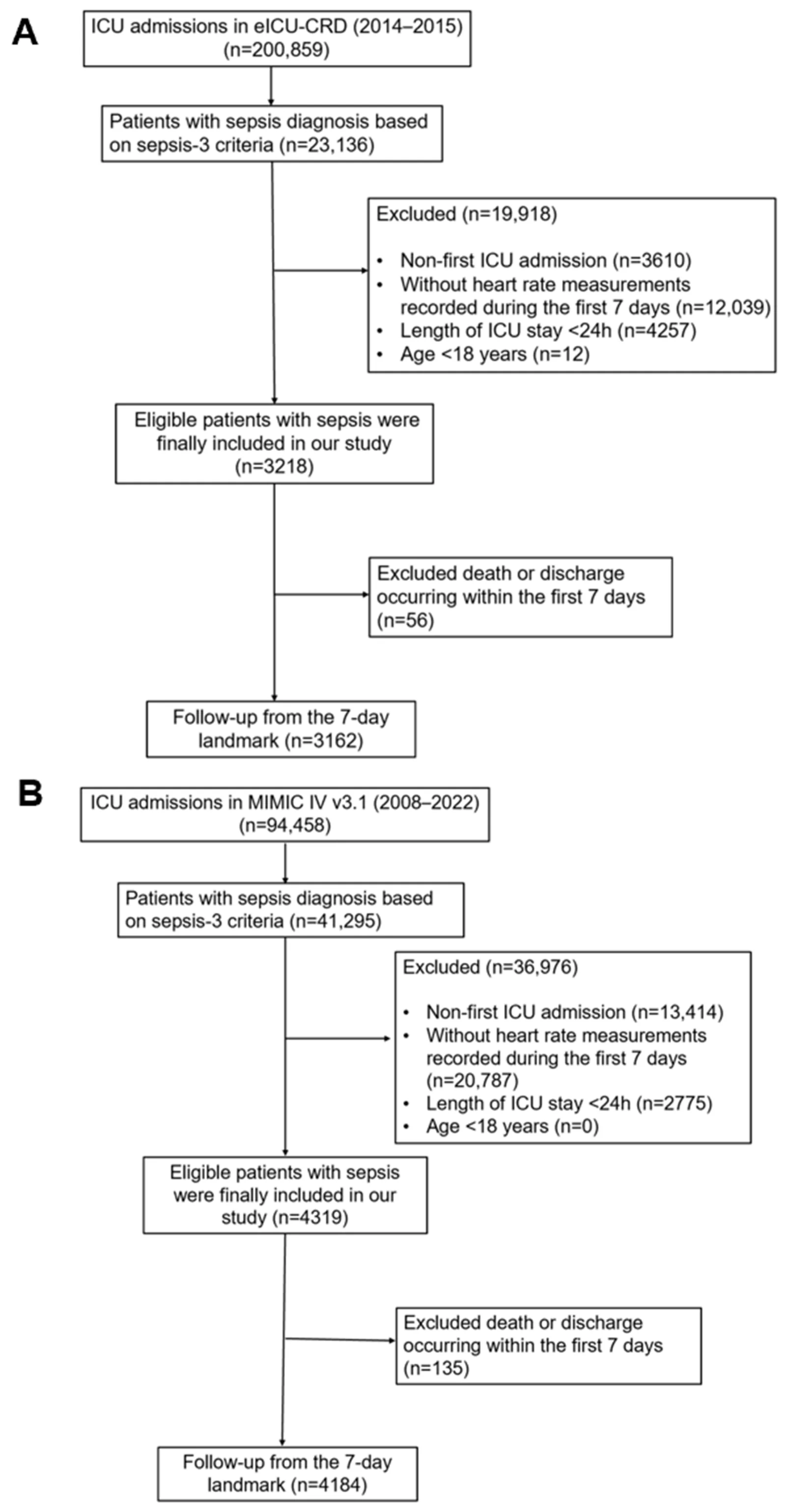
The research flow chart of patient inclusion procedure. Patient selection process for the (**A**) eICU-CRD cohort and the (**B**) MIMIC-IV cohort.

**Figure 2:**
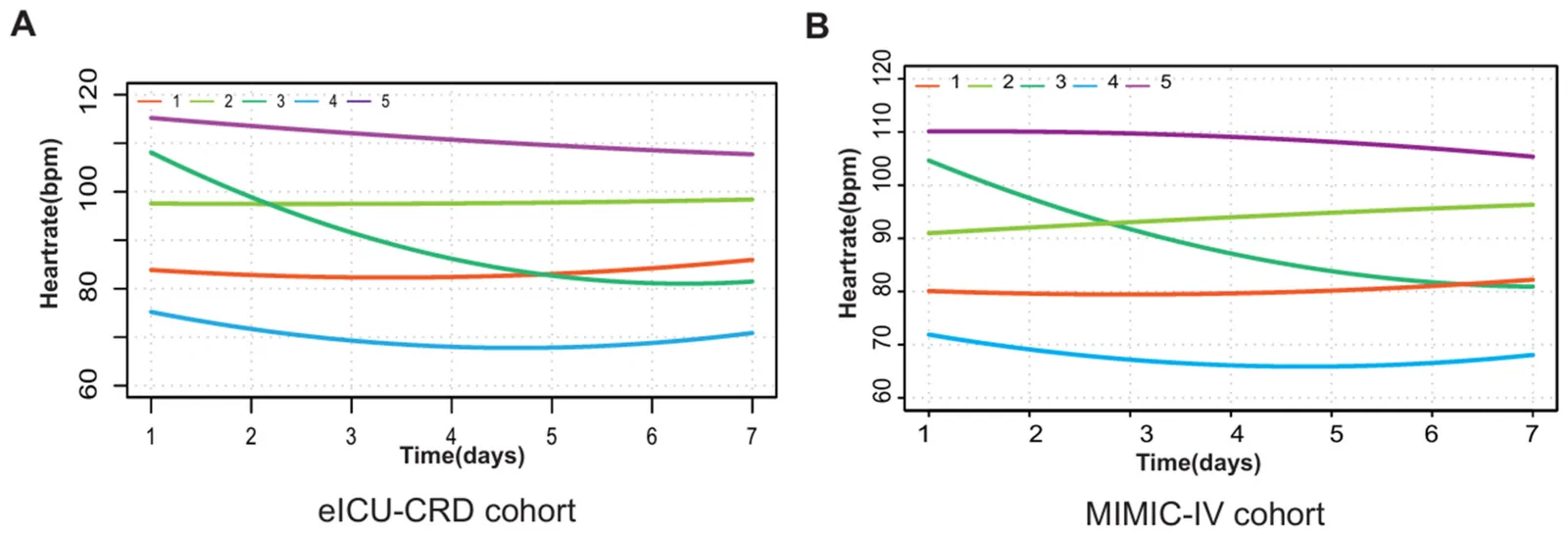
Heart rate trajectories over the first 7 days of ICU admission in sepsis patients. Five heart-rate trajectories were identified in the (**A**) eICU-CRD cohort and the (**B**) MIMIC-IV cohort, respectively. The *x*-axis indicates time in days from ICU admission, and the *y*-axis indicates heart rate (bpm).

A total of 7537 sepsis patients were included in this study, comprising 3218 patients in the eICU cohort and 4319 patients in the MIMIC-IV cohort (Figure 1). GBTM was applied to characterize heart rate dynamics during the first 7 days in the ICU. We fitted 1–6 class group-based trajectory models in both eICU-CRD and MIMIC-IV cohorts. Although the 6-class model had lower AIC/BIC, it produced subgroups with proportion < 10% and deteriorated classification performance. Compared with the 4-class model, the 5-class solution captured finer longitudinal heterogeneity of heart-rate patterns without generating unstable tiny subgroups and was therefore selected. (Figure 2; Tables S2 and S3). The average posterior probabilities for all five classes exceeded 0.70, indicating satisfactory model discrimination and reliability.

The five distinct heart rate trajectories were defined as follows:

Trajectory 1 (persistently low-stable trajectory): Low baseline heart rate (75–80 bpm), remained stable over 7 days with only a slight late increase.

Trajectory 2 (moderate-level gradually increasing trajectory): Moderate baseline heart rate (90–95 bpm) showed a gentle and continuous upward trend.

Trajectory 3 (high-baseline rapidly declining trajectory): High baseline heart rate (100–105 bpm) decreased rapidly within the first 3–4 days and then stabilized at a moderate level.

Trajectory 4 (low-level U-shaped trajectory): Lowest baseline heart rate (70–75 bpm), declined slowly to a trough within 4–5 days, followed by a mild rebound, maintaining an overall low level.

Trajectory 5 (persistently high trajectory): Highest baseline heart rate (110–115 bpm), remained persistently tachycardic with only a slight downward trend.

Violin plots of 24 h heart rate distribution across the 7-day period confirmed that temporal changes in both cohorts were highly consistent with trajectory definitions, supporting the stability of trajectory grouping (Figures S1 and S2).

### 3.2. Baseline Characteristics

Baseline characteristics, including demographics, vital signs, laboratory parameters, disease severity scores, and comorbidity burdens, differed significantly among the five trajectory groups in both cohorts (Tables S4 and S5). Patients in Traj-4 were significantly older, consistent with the physiological features of relatively lower heart rate and weaker circulatory compensation in elderly individuals. Traj-3 and Traj-4 were predominantly male, whereas the proportion of female patients was relatively higher in Traj-5. Patients in Traj-5 exhibited significantly higher respiratory rate, lactate level, white blood cell count, charlson comorbidity index, and SOFA score, as well as a higher prevalence of cardiac insufficiency, chronic renal insufficiency, and diabetes. By contrast, Traj-1 presented the most stable baseline status and the lowest comorbidity burden.

As shown in Table S6, in both the MIMIC IV and eICU-CRD cohorts, AKI patients were older, had higher disease severity (SOFA and Charlson comorbidity index, all *p* < 0.001), and had worse vital signs/laboratory parameters, including lactate, white blood cell counts, mean arterial pressure, hemoglobin, and platelet levels. The prevalence of comorbidities such as cardiac insufficiency, chronic renal insufficiency, diabetes, and hypertension was also significantly higher in AKI patients (all *p* < 0.001). Clinically, AKI patients had a substantially higher 28-day mortality rate, longer hospital stay, and greater use of renal replacement therapy compared with non-AKI patients (all *p* < 0.001; Table S7).

### 3.3. Associations Between Heart Rate Trajectories and Clinical Outcomes

There was a stable association between different heart rate trajectories and clinical outcomes of septic patients in both the eICU-CRD and MIMIC-IV cohorts (Table 1). Traj-5 also had the longest hospital stay (eICU-CRD cohort: 16.79 [11.14, 26.07] days; MIMIC-IV cohort: 20.73 [14.35, 29.83] days). Among the patients observed at day 7, the 7–28-day mortality was highest in Traj-2 (18.79%) for the eICU-CRD cohort and Traj-5 (18.78%) for the MIMIC-IV cohort. Among the patients who were alive, still in the ICU, and free of AKI at the 7-day landmark, the incidence of new-onset AKI in the Traj-5 was higher than that in other groups after the 7-day landmark (eICU-CRD cohort: 58.62% vs. 48.41–53.42%, *p* = 0.037; and MIMIC-IV cohort: 71.80% vs. 63.15–68.81%, *p* = 0.042), whereas Traj-4 presented the lowest new-onset AKI incidence (eICU-CRD cohort: 48.41%; MIMIC-IV cohort: 63.15%).

**Figure 3:**
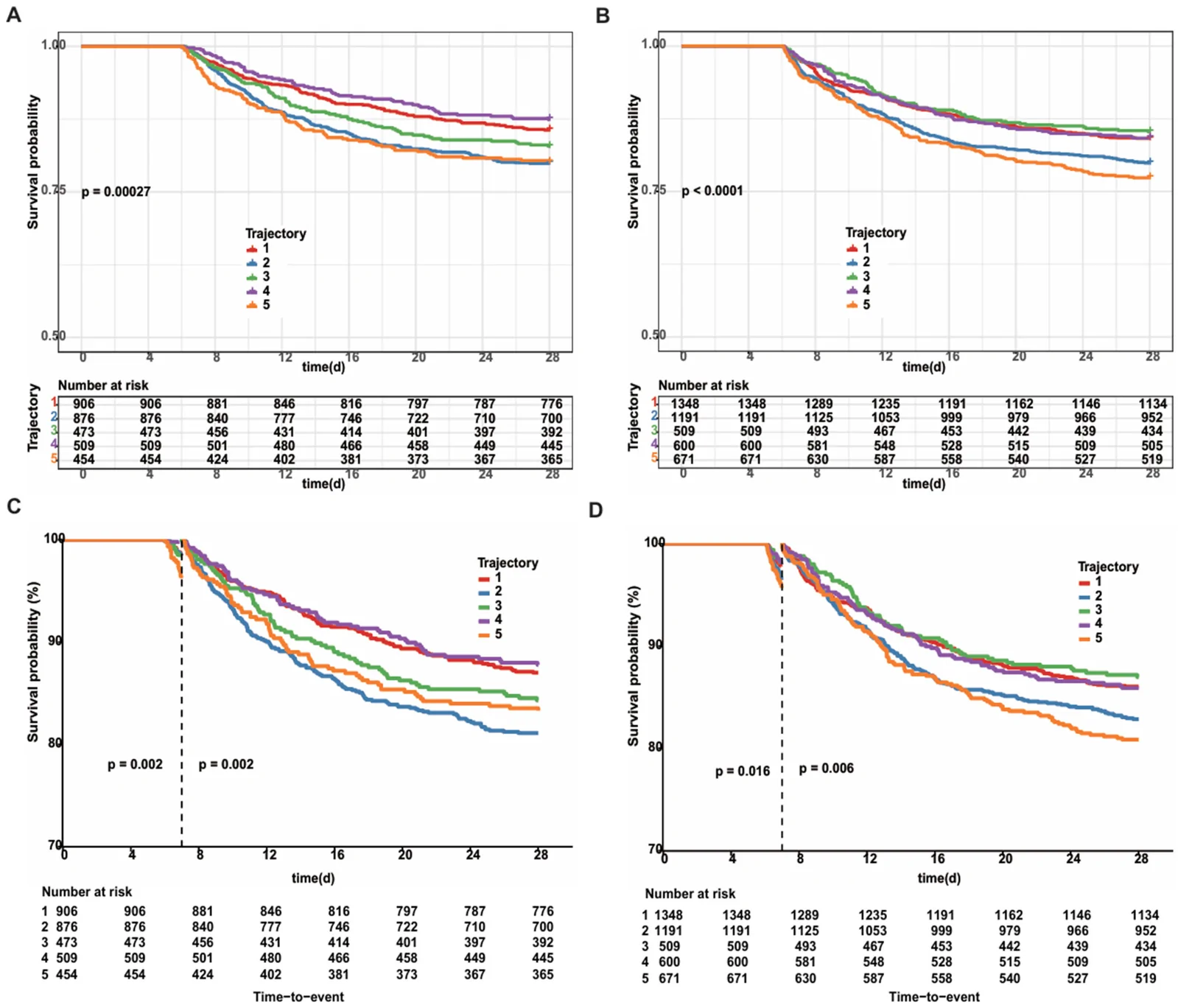
Kaplan–Meier survival curves for 28-day mortality among sepsis patients with different heart rate trajectories. (**A**,**B**) 28-day mortality in the eICU-CRD cohort and the MIMIC-IV cohort, respectively. (**C**,**D**) Landmark analysis (day 7) in the eICU-CRD cohort and the MIMIC-IV cohort, respectively.

Kaplan–Meier survival curves revealed significant differences in 28-day survival among trajectory groups in both cohorts (log-rank test: eICU-CRD, *p* = 0.00027; MIMIC-IV, *p* < 0.0001; Figure 3A,B). Patients in Traj-5 had the lowest survival probability and the steepest decline in survival, followed by those in Traj-2. Patients in Traj-1, Traj-3, and Traj-4 showed significantly higher and more gradual survival curves. Additionally, we performed landmark analysis with a landmark time of day 7 to account for immortal time bias (Figure 3C,D). Results showed that Traj-2 exhibited the lowest survival probabilities, with curves diverging from other groups after day 7 and declining most steeply over time in the eICU-CRD cohort (*p* = 0.002). However, in the MIMIC-IV cohort, Traj-5 showed the poorest survival outcomes, dropping below other trajectories after day 7 (*p* = 0.006).

Based on the 7-day landmark Cox regression, as shown in Table 2, with Traj-1 as the reference, after adjustment for age, gender, BMI, comorbidities, SOFA, lactate, mean arterial pressure, pacing treatment, mechanical ventilation, fever, bloodstream infection, severe arrhythmias, use of beta-blockers, sedatives, analgesics, antipyretics, vasoactive medications, and atrial fibrillation, Traj-2 and Traj-5 remained associated with an increased risk of 28-day mortality in the two cohorts after the 7-day landmark (eICU-CRD cohort: Traj-2 adjusted HR = 1.51 (95% CI: 1.18–1.92, *p* < 0.001)); Traj-5 adjusted HR = 1.57 (95% CI: 1.16–2.12, *p* = 0.003)). MIMIC-IV cohort: Traj-2 adjusted HR = 1.26 (95% CI: 1.03–1.55, *p* = 0.027); Traj-5 adjusted HR = 1.46 (95% CI:1.15–1.85, *p* = 0.002). No significant association with mortality was observed for Traj-3 and Traj-4.

### 3.4. Associations Between Heart Rate Trajectories and AKI

Multivariable logistic regression was used to explore the relationship between heart rate trajectories and AKI incidence after the 7-day landmark (Table 3). After adjustment for age, gender, disease severity, comorbidities, clinical complications, and intensive care interventions, Traj-5 was associated with a higher risk of AKI compared with Traj-1 in both cohorts after the 7-day landmark (all *p* < 0.05). (adjusted OR in eICU-CRD cohort: 1.82 [95% CI, 1.34–2.48], *p* < 0.001; and adjusted OR in MIMIC-IV cohort: 1.58 [95% CI, 1.05–2.38], *p* = 0.03). No significant associations with AKI were detected for Traj-2, Traj-3 and Traj-4. These results are consistent with pathophysiological mechanisms linking persistent tachycardia to reduced renal perfusion, sustained sympathetic activation, and amplified inflammatory stress.

### 3.5. Linear Associations Between Heart Rate and 28-Day Mortality

**Figure 4:**
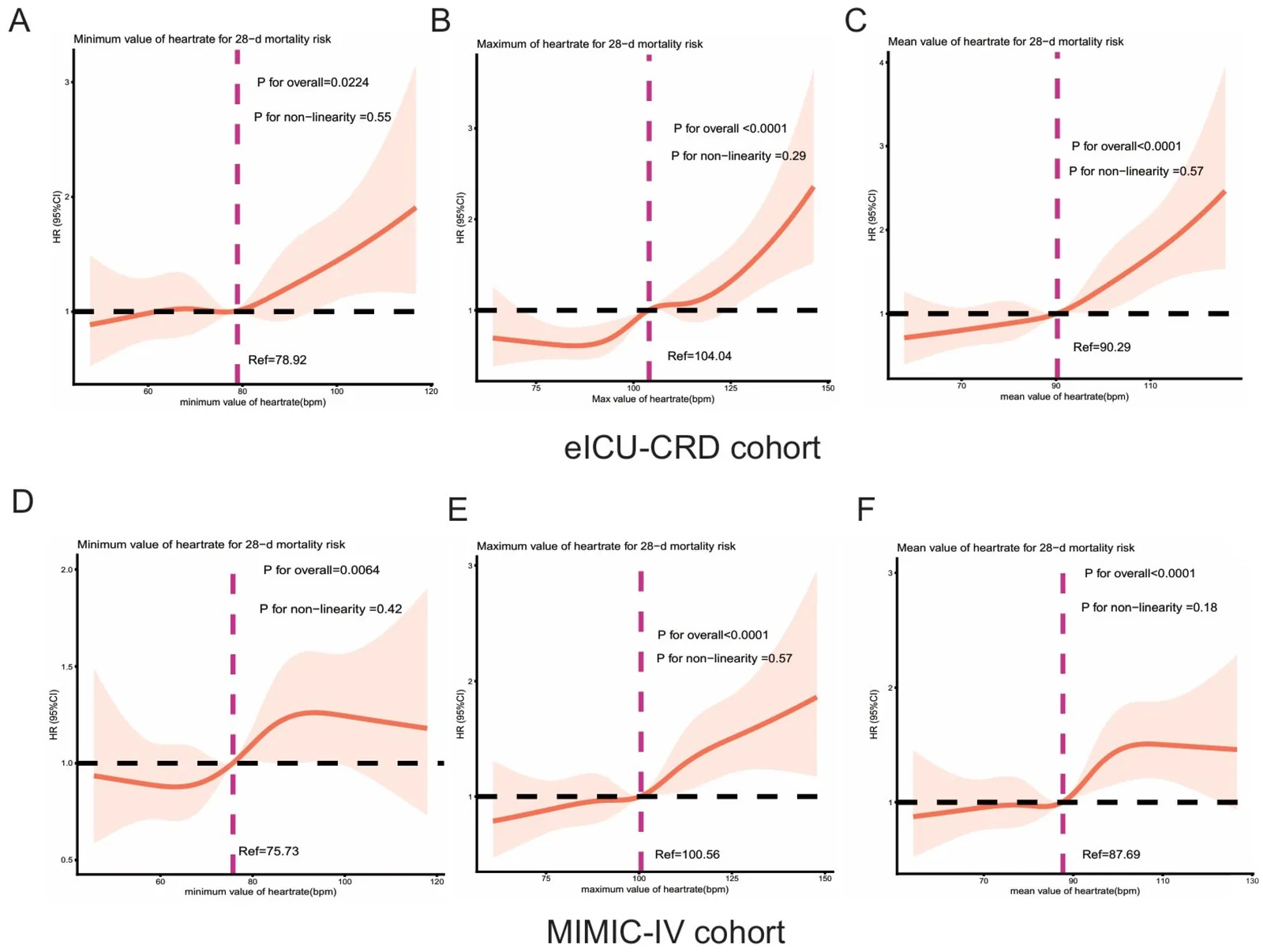
Restricted cubic spline (RCS) curve showing the association between minimum, maximum, and mean heart rate and 28-day mortality risk in sepsis patients. (**A**) minimum heart rate, (**B**) maximum heart rate, (**C**) mean heart rate and 28-day mortality risk in the eICU-CRD cohort; (**D**) minimum heart rate, (**E**) maximum heart rate, and (**F**) mean heart rate and 28-day mortality risk in the MIMIC-IV cohort. HR, hazard ratio; CI, confidence interval.

RCS analysis showed that in both the eICU-CRD and MIMIC-IV cohorts, the minimum, maximum, and mean heart rates were all significantly and linearly positively correlated with 28-day mortality risk (Figure 4; all *p* for overall < 0.05). After exceeding their respective reference values, 28-day mortality risk increased as heart rate rose. No significant non-linear associations were detected (Figure 4; all *p* for non-linearity > 0.05).

### 3.6. Subgroup Analyses

Subgroup analyses were conducted stratified by age (≥65 vs. <65 years), BMI (<25, 25–30, ≥30 kg/m^2^), Charlson Comorbidity Index, SOFA score, gender, cardiac insufficiency, chronic renal insufficiency, mechanical ventilation, vasopressor use, renal replacement therapy, and AKI status (Figures S3 and S4). With Traj-1 as the reference, the increased 28-day mortality risk associated with Traj-5 remained stable and consistent across all subgroups, with no significant interaction effects (all *p* for interaction > 0.05; Figures S3 and S4). Traj-3 and Traj-4 showed no significant association with mortality in any subgroup, confirming the robustness and generalizability of the main findings.

### 3.7. Sensitivity Analysis

Sensitivity analyses were sequentially performed by excluding patients with severe arrhythmias, the use of beta-blockers, pacing treatment, and atrial fibrillation in separate models (Table S8). Consistent findings were observed across the eICU and MIMIC-IV cohorts. After these exclusions, Traj-2 and Traj-5 remained associated with elevated 28-day mortality, with Traj-1 as the reference in most adjusted models. No consistent significant associations with 28-day mortality were detected for Traj-3 and Traj-4 in either database after the sequential exclusions. Additionally, considering the heterogeneity in follow-up duration across trajectory subgroups after day 7, the Cox proportional-hazards model was applied to investigate the association between heart-rate trajectories and incident AKI following the 7-day landmark (Table S9). Results demonstrated that Traj-5 was associated with elevated new-onset AKI risk in both eICU-CRD (adjusted HR = 1.26 (95% CI: 1.10–1.45, *p* < 0.001)) and MIMIC-IV cohorts (adjusted HR = 1.11 (95% CI: 1.01–1.24, *p* = 0.046) after full adjustment. Collectively, these sensitivity results were directionally consistent with the primary analysis.

## 4. Discussion

Based on two independent cohorts from the MIMIC-IV and eICU-CRD databases, this study employed group-based trajectory modeling (GBTM) to characterize dynamic heart rate patterns during the first 7 days after ICU admission among 7537 patients with sepsis and identified five heart rate trajectories. The results demonstrated that persistently high heart rate trajectory (Traj-5) was associated with increased risks of 28-day mortality and new-onset AKI among patients observed after day 7. The associations remained consistent across subgroups stratified by age, comorbidities, and disease severity. Additionally, sepsis patients complicated by AKI exhibited more severe baseline conditions and circulatory disturbances, and the association between heart rate trajectories and adverse outcomes remained significant in the AKI subgroup.

Heart rate serves as a crucial marker of early stress response in sepsis and also acts as a key indicator reflecting circulatory compensation, inflammatory burden, and tissue perfusion [19]. The baseline level and dynamic change patterns are closely associated with the risk of mortality in septic patients [20]. Excessive sympathetic nervous system activation is the core cause of sepsis-induced myocardial depression and arrhythmia. Myocardial depression, calcium overload, mitochondrial dysfunction, and microcirculatory disturbance all manifest as the sustained elevated or unstable heart rate [21,22,23,24]. Compared with normal heart rate, elevated heart rate is significantly correlated with mortality, in-hospital death, and long-term prognosis [25]. Changes in heart rate can serve as an early warning sign of infection risk before patients develop the typical critical symptoms of sepsis, providing a crucial basis for early clinical intervention and improved patient outcomes [25,26,27,28,29]. A retrospective study found that early HR fluctuation was associated with an increased risk of death among patients with sepsis [30]. Previous studies have found that patients with progressively increasing heart rates after admission have a significantly higher risk of death, whereas long-term treatment with beta-blockers can improve survival outcomes in patients with sepsis [31,32,33]. Different heart rate trajectories reflect the severity of pathological injury and varied responses to treatment. Previous meta-analyses have demonstrated a clear threshold effect for heart rate control, and targeting a heart rate of 65–70 bpm reduces mortality in patients with coronary heart disease and heart failure [34]. Consistent with these findings, this study showed that Traj-5 was associated with the highest risks of 28-day mortality and AKI, which also supports our restricted cubic spline results that mortality risk increases significantly once heart rate exceeds a certain threshold.

Arrhythmias are closely associated with the onset and progression of AKI [35]. By shortening cardiac diastole, reducing renal perfusion pressure, activating the sympathetic nervous system, and exacerbating inflammatory response and oxidative stress, tachycardia decreases renal blood flow and induces ischemic injury to renal tubules, ultimately increasing the risk of AKI [36]. Persistently elevated heart rate is the key indicator of stress in critically ill patients, inadequate tissue perfusion, and poor prognosis [37,38,39]. A recent study showed that prolonged elevated heart rate was significantly associated with 90-day mortality, in-hospital mortality, and renal function deterioration in patients with sepsis-associated AKI and could serve as an effective prognostic indicator [40]. Our findings support and expand these conclusions. Patients with persistently elevated heart rate trajectory (Traj-5) exhibited the lowest 28-day survival rate and markedly higher risk of AKI. This association was verified in two independent cohorts (eICU and MIMIC-IV databases), demonstrating robust and reproducible results. It also highlights the importance of monitoring longitudinal heart rate trajectory changes in the care of septic patients, which helps clinicians identify septic patients with hemodynamic instability at an early stage.

In clinical practice, our findings provide important insights that heart rate is probably a marker of disease severity such as shock, inflammation, fever, catecholamines, arrhythmias, and treatment intensity. When managing septic patients, beyond paying attention to the absolute value of heart rate, greater emphasis should be placed on its dynamic fluctuations and overall stability. Distinct heart rate trajectories may help identify patients who demonstrate physiological responses to treatment. Traj-2 is characterized by a moderate initial heart rate followed by progressive elevation. indicating occult clinical deterioration in septic patients. The gradual rise may result from shock progression, secondary infection, or worsening myocardial injury [41]. Even without early hemodynamic collapse, persistent inflammation drives sympathetic activation and irreversible damage [42]. Traj-3 presents marked tachycardia at baseline, followed by rapid decline of heart rate. This favorable trend may be closely associated with effective clinical management. Rapid recovery primarily may benefit from successful infection source control, adequate and timely fluid resuscitation, and early individualized titration of vasopressors, reflecting reversal of systemic stress and hemodynamic disturbance [43]. Traj-4 shows a typical low U-shaped pattern, with a relatively low overall heart rate. Contributing factors include milder inflammation, preserved cardiac function reducing compensatory demand, and long-term beta-blocker use [44]. Additionally, elderly patients generally present with a lower intrinsic heart rate and blunted response to stress stimuli [45]. Traj-5 is defined as sustained tachycardia, which arises from multiple pathophysiological mechanisms, including persistent sympathetic overactivation, continuous inflammatory cytokine signaling, unresolved circulatory shock [46], impaired ventriculo-arterial coupling, compensatory tachycardia caused by reduced stroke volume [47], sepsis-induced cardiomyopathy, exogenous catecholamine administration, and persistent fever, as well as pain and agitation. In a sense, sustained tachycardia may be a sign of a worsening condition. Notably, sustained tachycardia shortens diastolic filling, increases myocardial oxygen consumption, impairs coronary perfusion [48], and disrupts renal blood flow, forming a self-perpetuating cardio-renal vicious cycle that exacerbates organ injury and worsens outcomes.

This study has the following limitations. Firstly, this retrospective study only enrolled patients with complete heart rate records during the first 7 days of ICU admission, which led to inevitable immortal time bias and survivor bias. The highest-risk heart rate trajectories may not be identified. Secondly, there exists reverse causality between heart rate trajectories and acute kidney injury. Abnormal heart rate may promote the occurrence of AKI, while impaired renal function can also conversely alter heart rate and hemodynamic status, making it difficult to distinguish the exact causal sequence. Thirdly, in septic patients, heart rate abnormalities are frequently complicated by diverse cardiac electrical disturbances. The precise identification of specific arrhythmia types—including life-threatening ventricular arrhythmias and ST-segment elevation myocardial infarction—necessitates 12-lead electrocardiography for accurate diagnosis [48,49,50,51]. Fourthly, given the unavailability of complete data on AKI occurrence after patient discharge, competing-risk adjustment for the competing events was not performed. Therefore, future studies are needed to validate the association between heart-rate trajectories and new-onset AKI. Furthermore, comprehensive data of medications such as vasoactive agents (including dose titration and temporal changes in administration) could not be fully ascertained. The covariates about intensive care interventions included in Model 3 were collected within the initial 7-day ICU monitoring window. Several indicators (such as mechanical ventilation duration, vasoactive medications, sedatives, analgesics, fever, bloodstream infection, severe arrhythmias, etc.) may develop dynamically during the 7-day observation period or even emerge after the trajectory window is completed. They may act as intermediate mediators reflecting disease progression or markers of clinical treatment intensity. These potential confounding variables could not be completely adjusted for given the inherent limitations of this retrospective study design. Lastly, this study focused exclusively on adult patients with sepsis; therefore, the findings cannot be directly extrapolated to other critically ill populations.

## 5. Conclusions

This study revealed that early dynamic heart rate trajectories in septic patients are associated with adverse clinical outcomes. Among patients observed at day 7, compared with patients with persistently low heart rate trajectories, those with persistently high heart rate trajectories were associated with higher risks of 28-day mortality and new-onset AKI after the landmark. This association remained directionally consistent after multivariable adjustment across two cohorts. However, residual confounding may not be fully excluded. These findings support intensified clinical surveillance, as heart rate can provide valuable information for prognostic association and risk stratification.

## Figures and Tables

**Table 1:** Clinical outcomes between different trajectories of heart rate.

	Overall	Traj-1	Traj-2	Traj-3	Traj-4	Traj-5	*p* Value
eICU-CRD cohort							
Length of ICU stay (days)	8.79 [6.21, 13.46]	8.69 [6.13, 13.63]	8.83 [6.33, 13.06]	8.50 [5.92, 12.29]	8.33 [5.67, 12.92]	10.00 [6.93, 15.25]	<0.001
Length of hospital stay (days)	15.17 [10.63, 22.38]	15.06 [10.58, 21.83]	15.42 [10.96, 23.09]	14.96 [10.71, 21.38]	14.17 [9.75, 20.75]	16.79 [11.14, 26.07]	<0.001
7–28-day mortality, *n* (%)	485 (15.34)	116 (13.00)	162 (18.79)	72 (15.52)	62 (12.23)	73 (16.70)	0.003
New-onset AKI, *n* (%)	1535 (52.18)	420 (51.09)	414 (51.56)	234 (53.42)	229 (48.41)	238 (58.62)	0.037
MIMIC IV cohort							
Length of ICU stay (days)	11.27 [8.23, 16.82]	11.00 [8.16, 16.16]	11.51 [8.22, 17.03]	10.89 [8.11, 16.22]	11.75 [8.48, 16.86]	11.64 [8.33, 17.34]	0.194
Length of hospital stay (days)	18.77 [13.19, 28.02]	17.95 [13.02, 26.73]	19.20 [13.17, 28.24]	19.38 [13.37, 29.28]	17.78 [12.91, 26.71]	20.73 [14.35, 29.83]	<0.001
7–28-day mortality, *n* (%)	640 (15.30)	181 (13.76)	192 (16.78)	64 (12.85)	83 (14.12)	120 (18.78)	0.010
New-onset AKI, *n* (%)	2526 (67.96)	787 (67.44)	697 (68.81)	298 (68.04)	329 (63.15)	415 (71.80)	0.042

Note: Data are shown as median [IQR] for continuous variables and n (%) for categorical variables. Abbreviations: RRT, renal replacement therapy; AKI, acute kidney injury.

**Table 2:** Multivariable Cox regression analysis used to evaluate the associations between different heart rate trajectories and 28-day mortality in patients with sepsis after the 7-day landmark.

Trajectory Model	Unadjusted Model		Model 1		Model 2		Model 3	
	HR (95% CI)	*p* Value	HR (95% CI)	*p* Value	HR (95% CI)	*p* Value	HR (95% CI)	*p* Value
eICU-CRD cohort								
Traj-1	Reference		Reference		Reference		Reference	
Traj-2	1.51 (1.19–1.91)	<0.001	1.56 (1.23–1.98)	<0.001	1.55 (1.22–1.98)	<0.001	1.51 (1.18–1.92)	<0.001
Traj-3	1.21 (0.90–1.63)	0.197	1.25 (0.93–1.68)	0.135	1.26 (0.94–1.69)	0.121	1.25 (0.93–1.68)	0.138
Traj-4	0.94 (0.69–1.28)	0.683	0.91 (0.67–1.24)	0.564	0.92 (0.67–1.25)	0.583	0.92 (0.68–1.26)	0.609
Traj-5	1.33 (0.99–1.78)	0.058	1.50 (1.12–2.02)	0.007	1.53 (1.13–2.06)	0.005	1.57 (1.16–2.12)	0.003
MIMIC IV cohort								
Traj-1	Reference		Reference		Reference		Reference	
Traj-2	1.24 (1.01–1.52)	0.038	1.29 (1.05–1.58)	0.014	1.29 (1.05–1.58)	0.014	1.26 (1.03–1.55)	0.027
Traj-3	0.93 (0.70–1.23)	0.600	0.98 (0.73–1.30)	0.866	0.97 (0.73–1.28)	0.807	0.94 (0.71–1.26)	0.695
Traj-4	1.03 (0.79–1.33)	0.843	1.03 (0.79–1.33)	0.837	1.04 (0.80–1.35)	0.748	1.06 (0.81–1.38)	0.665
Traj-5	1.39 (1.11–1.75)	0.005	1.63 (1.29–2.06)	<0.001	1.53 (1.21–1.93)	<0.001	1.46 (1.15–1.85)	0.002

Adjusted Model 1: Adjustments were made for age, BMI, gender. Adjusted Model 2: Building on adjusted Model 1, further adjustments were incorporated for cardiac insufficiency, chronic hepatic insufficiency, chronic renal insufficiency, diabetes, chronic pulmonary disease, malignant cancer, organ transplant, and hypertension. Adjusted Model 3: Building on adjusted Model 2, further adjustments were incorporated for SOFA, lactate, mean arterial pressure, pacing treatment, mechanical ventilation, fever, bloodstream infection, severe arrhythmias, use of beta-blockers, sedatives, analgesics, antipyretics, vasoactive medications, and atrial fibrillation. HR: Hazard ratio; CI: confidence interval.

**Table 3:** Association between heart rate trajectory and AKI occurrence after the 7-day landmark.

Trajectory Model	Unadjusted Model		Model1		Model2		Model3	
	OR (95%CI)	*p* Value	OR (95%CI)	*p* Value	OR (95%CI)	*p* Value	OR (95%CI)	*p* Value
eICU-CRD cohort								
Traj-1	Reference		Reference		Reference		Reference	
Traj-2	0.93 (0.75–1.16)	0.532	0.98 (0.79–1.22)	0.842	0.99 (0.79–1.24)	0.951	0.96 (0.76–1.21)	0.748
Traj-3	0.99 (0.76–1.29)	0.951	1.00 (0.77–1.30)	0.997	1.00 (0.76–1.30)	0.984	0.99 (0.75–1.30)	0.915
Traj-4	0.88 (0.69–1.13)	0.317	0.84 (0.65–1.08)	0.179	0.79 (0.61–1.02)	0.069	0.77 (0.59–1.00)	0.054
Traj-5	1.46 (1.10–1.95)	0.010	1.61 (1.20–2.16)	0.001	1.67 (1.24–2.25)	<0.001	1.82 (1.34–2.48)	<0.001
MIMIC IV cohort								
Traj-1	Reference		Reference		Reference		Reference	
Traj-2	1.17 (0.87–1.58)	0.305	1.24 (0.91–1.69)	0.169	1.25 (0.91–1.70)	0.170	1.26 (0.92–1.73)	0.157
Traj-3	1.40 (0.92–2.13)	0.115	1.46 (0.95–2.26)	0.085	1.42 (0.92–2.20)	0.117	1.52 (0.96–2.39)	0.072
Traj-4	0.82 (0.59–1.15)	0.255	0.75 (0.53–1.06)	0.102	0.77 (0.54–1.09)	0.143	0.75 (0.53–1.08)	0.127
Traj-5	1.46 (1.00–2.15)	0.051	1.71 (1.15–2.55)	0.008	1.61 (1.08–2.41)	0.020	1.58 (1.05–2.38)	0.030

Adjusted Model 1: Adjustments were made for age, BMI, and gender. Adjusted Model 2: Building on adjusted Model 1, further adjustments were incorporated for cardiac insufficiency, chronic hepatic insufficiency, chronic renal insufficiency, diabetes, chronic pulmonary disease, malignant cancer, organ transplant, and hypertension. Adjusted Model 3: Building on adjusted Model 2, further adjustments were incorporated for SOFA, lactate, mean arterial pressure, pacing treatment, mechanical ventilation, fever, bloodstream infection, severe arrhythmias, use of beta-blockers, sedatives, analgesics, antipyretics, vasoactive medications, and atrial fibrillation. OR, Odds Ratio; CI, confidence interval.

## Data Availability

The original contributions presented in this study are included in the article/Supplementary Materials; further inquiries can be directed to the corresponding author.

## Supplementary Materials

The following supporting information can be downloaded at https://www.mdpi.com/article/10.3390/jcm15176660/s1, Figure S1: Dynamic changes in heart rate levels across five trajectories over seven 24 h periods based on eICU-CRD cohort; Figure S2: Dynamic changes in heart rate levels across five trajectories over seven 24 h periods based on MIMIC-IV cohort; Figure S3: Forest plot of subgroup analyses for the association between heart rate trajectory groups and 28-day mortality based on eICU-CRD cohort; Figure S4: Forest plot of subgroup analyses for the association between heart rate trajectory groups and 28-day mortality based on MIMIC IV cohort. Table S1: Proportion of missing values for continuous variables in eICU cohort and MIMIC-IV cohort; Table S2: Group-based trajectory modeling for choosing the best number of phenotypes based on MIMIC IV cohort; Table S3: Group-based trajectory modeling for choosing the best number of phenotypes based on eICU-CRD cohort; Table S4: Baseline characteristics of included participants in eICU-CRD cohort; Table S5: Baseline characteristics of included participants in MIMIC IV cohort; Table S6: Baseline characteristics between AKI and non-AKI patients in the MIMIC-IV and eICU-CRD cohorts; Table S7: Clinical outcomes between AKI and Non-AKI patients in the MIMIC-IV and eICU-CRD cohorts; Table S8: Sensitivity analyses examining the associations between heart rate trajectory and 28-day mortality after excluding patients with severe arrhythmias, use of beta-blockers, pacing treatment and atrial fibrillation sequentially; Table S9: Cox proportional hazards models examining the associations between heart rate trajectory and the risk of AKI after the 7-day landmark.

## References

[1] Chiu C., Legrand M. (2021). Epidemiology of Sepsis and Septic Shock. Curr. Opin. Anaesthesiol..

[2] Singer M., Deutschman C.S., Seymour C.W., Shankar-Hari M., Annane D., Bauer M., Bellomo R., Bernard G.R., Chiche J.-D., Coopersmith C.M. (2016). The Third International Consensus Definitions for Sepsis and Septic Shock (Sepsis-3). JAMA.

[3] Rhee C., Dantes R., Epstein L., Murphy D.J., Seymour C.W., Iwashyna T.J., Kadri S.S., Angus D.C., Danner R.L., Fiore A.E. (2017). Incidence and Trends of Sepsis in US Hospitals Using Clinical vs Claims Data, 2009–2014. JAMA.

[4] Martin-Loeches I., Singer M., Leone M. (2024). Sepsis: Key Insights, Future Directions, and Immediate Goals. A Review and Expert Opinion. Intensive Care Med..

[5] Pecchiari M., Pontikis K., Alevrakis E., Vasileiadis I., Kompoti M., Koutsoukou A. (2021). Cardiovascular Responses During Sepsis. Compr. Physiol..

[6] Leone M., Russell L., Cecconi M., Khanna A.K., Pirracchio R., Bauer M., Rello J. (2025). Hemodynamic Failure during Sepsis: What Clinicians and Researchers Must Know. Anaesth. Crit. Care Pain Med..

[7] Hato T., Dagher P.C. (2025). Molecular Mechanisms of Sepsis-Associated Acute Kidney Injury. J. Am. Soc. Nephrol..

[8] Vincent J.-L. (2022). Current Sepsis Therapeutics. EBioMedicine.

[9] Antonucci E., Garcia B., Legrand M. (2024). Hemodynamic Support in Sepsis. Anesthesiology.

[10] Kiernan E., Zelnick L.R., Khader A., Coston T.D., Bailey Z.A., Speckmaier S., Lo J.J., Siew E.D., Sathe N.A., Kestenbaum B.R. (2025). Molecular Phenotyping of Sepsis and Differential Response to Fluid Resuscitation. Am. J. Respir. Crit. Care Med..

[11] Arbo J.E., Lessing J.K., Ford W.J.H., Clark S., Finkelsztein E., Schenck E.J., Sharma R., Heerdt P.M. (2020). Heart Rate Variability Measures for Prediction of Severity of Illness and Poor Outcome in ED Patients with Sepsis. Am. J. Emerg. Med..

[12] Ning Y.-L., Li W.-J., Lu X., Zhang Y., Zhang J.-W., Zhou J.-H. (2024). Association between Heart Rate and Mortality in Patients with Septic Shock: An Analysis Revealed by Time Series Data. BMC Infect. Dis..

[13] Shen Y., Wang J., Cao Q., Wu Y., Wang Q., Wang N., Shao M. (2026). Maximum Heart Rate and Mortality in Sepsis Patients: A Retrospective Cohort Study. Intern. Emerg. Med..

[14] Pollard T.J., Johnson A.E.W., Raffa J.D., Celi L.A., Mark R.G., Badawi O. (2018). The eICU Collaborative Research Database, a Freely Available Multi-Center Database for Critical Care Research. Sci. Data.

[15] Johnson A.E.W., Bulgarelli L., Shen L., Gayles A., Shammout A., Horng S., Pollard T.J., Hao S., Moody B., Gow B. (2023). MIMIC-IV, a Freely Accessible Electronic Health Record Dataset. Sci. Data.

[16] Hofford M.R., Yu S.C., Johnson A.E.W., Lai A.M., Payne P.R.O., Michelson A.P. (2022). OpenSep: A Generalizable Open Source Pipeline for SOFA Score Calculation and Sepsis-3 Classification. JAMIA Open.

[17] Khwaja A. (2012). KDIGO Clinical Practice Guidelines for Acute Kidney Injury. Nephron Clin. Pract..

[18] Nagin D.S., Jones B.L., Elmer J. (2024). Recent Advances in Group-Based Trajectory Modeling for Clinical Research. Annu. Rev. Clin. Psychol..

[19] Adam J., Rupprecht S., Künstler E.C.S., Hoyer D. (2023). Heart Rate Variability as a Marker and Predictor of Inflammation, Nosocomial Infection, and Sepsis—A Systematic Review. Auton. Neurosci..

[20] Demirjian S., Bakaeen F., Tang W.H.W., Donaldson C., Taliercio J., Huml A., Gadegbeku C.A., Gillinov A.M., Insler S.D. (2024). Hemodynamic Determinants of Cardiac Surgery-Associated Acute Kidney Injury. Crit. Care Explor..

[21] Sato R., Sanfilippo F., Lanspa M., Duggal A., Dugar S. (2025). Sepsis-Induced Cardiomyopathy: Mechanism, Prevalence, Assessment, Prognosis, and Management. Chest.

[22] Pamporis K., Karakasis P., Pantelidaki A., Goutis P.A., Grigoriou K., Theofilis P., Katsaouni A., Botis M., Karanikola A.-E., Milaras N. (2025). Sepsis-Induced Cardiomyopathy and Cardiac Arrhythmias: Pathophysiology and Implications for Novel Therapeutic Approaches. Biomedicines.

[23] Zakynthinos G.E., Tsolaki V., Oikonomou E., Vavouranakis M., Siasos G., Zakynthinos E. (2023). New-Onset Atrial Fibrillation in the Critically Ill COVID-19 Patients Hospitalized in the Intensive Care Unit. J. Clin. Med..

[24] Leibovici L., Gafter-Gvili A., Paul M., Almanasreh N., Tacconelli E., Andreassen S., Nielsen A., Frank U., Cauda R., TREAT Study Group (2007). Relative Tachycardia in Patients with Sepsis: An Independent Risk Factor for Mortality. QJM Int. J. Med..

[25] de Castilho F.M., Ribeiro A.L.P., Nobre V., Barros G., de Sousa M.R. (2018). Heart Rate Variability as Predictor of Mortality in Sepsis: A Systematic Review. PLoS ONE.

[26] van Wijk R.J., Quinten V.M., van Rossum M.C., Bouma H.R., Ter Maaten J.C. (2023). Predicting Deterioration of Patients with Early Sepsis at the Emergency Department Using Continuous Heart Rate Variability Analysis: A Model-Based Approach. Scand. J. Trauma Resusc. Emerg. Med..

[27] Barnaby D.P., Fernando S.M., Herry C.L., Scales N.B., Gallagher E.J., Seely A.J.E. (2019). Heart Rate Variability, Clinical and Laboratory Measures to Predict Future Deterioration in Patients Presenting with Sepsis. Shock.

[28] Liu N., Chee M.L., Foo M.Z.Q., Pong J.Z., Guo D., Koh Z.X., Ho A.F.W., Niu C., Chong S.-L., Ong M.E.H. (2021). Heart Rate n-Variability (HRnV) Measures for Prediction of Mortality in Sepsis Patients Presenting at the Emergency Department. PLoS ONE.

[29] Fairchild K.D., O’Shea T.M. (2010). Heart Rate Characteristics: Physiomarkers for Detection of Late-Onset Neonatal Sepsis. Clin. Perinatol..

[30] He J., Yang J., Liu J. (2023). Early Heart Rate Fluctuation and Outcomes in Critically Ill Patients with Sepsis: A Retrospective Cohort Study of the MIMIC-IV Database. Heliyon.

[31] Huang P.-Y., Liu T.-H., Wu J.-Y., Tsai Y.-W., Hsu W.-H., Chuang M.-H., Tang H.-J., Lai C.-C. (2026). Effect of Ultrashort-Acting β-Blocker on the Mortality of Patients with Sepsis or Septic Shock: A Systematic Review and Trial Sequential Meta-Analysis of Randomized Controlled Trials. Intensive Crit. Care Nurs..

[32] Guz D., Buchritz S., Guz A., Ikan A., Babich T., Daitch V., Gafter-Gvili A., Leibovici L., Avni T. (2021). β-Blockers, Tachycardia, and Survival Following Sepsis: An Observational Cohort Study. Clin. Infect. Dis..

[33] Kakihana Y., Nishida O., Taniguchi T., Okajima M., Morimatsu H., Ogura H., Yamada Y., Nagano T., Morishima E., Matsuda N. (2020). Efficacy and Safety of Landiolol, an Ultra-Short-Acting β1-Selective Antagonist, for Treatment of Sepsis-Related Tachyarrhythmia (J-Land 3S): A Multicentre, Open-Label, Randomised Controlled Trial. Lancet Respir. Med..

[34] Sanidas E., Böhm M., Oikonomopoulou I., Dinopoulou P., Papadopoulos D., Michalopoulou H., Tsioufis K., Mancia G., Thomopoulos C. (2025). Heart Rate-Lowering Drugs and Outcomes in Hypertension and/or Cardiovascular Disease: A Meta-Analysis. Eur. Heart J..

[35] Genovesi S., Regolisti G., Burlacu A., Covic A., Combe C., Mitra S., Basile C., Bartolucci C., The EuDial Working Group of ERA (2023). The Conundrum of the Complex Relationship between Acute Kidney Injury and Cardiac Arrhythmias. Nephrol. Dial. Transplant..

[36] Schefold J.C., Filippatos G., Hasenfuss G., Anker S.D., von Haehling S. (2016). Heart Failure and Kidney Dysfunction: Epidemiology, Mechanisms and Management. Nat. Rev. Nephrol..

[37] Hayase N., Yamamoto M., Asada T., Isshiki R., Doi K. (2024). Tachycardia and Acute Kidney Injury among Critically Ill Patients with Sepsis: A Prospective Observational Study. Blood Purif..

[38] Kim S.G., Yun D., Kim J., Lee J., Kang M.W., Kim Y.C., Kim D.K., Oh K.-H., Joo K.W., Koo H. (2023). Risk of Ventricular Tachycardia and Its Outcomes in Patients Undergoing Continuous Renal Replacement Therapy due to Acute Kidney Injury. Kidney Res. Clin. Pract..

[39] Kuo L., Muser D., Shirai Y., Lin A., Liang J., Schaller R.D., Hyman M., Kumareswaran R., Arkles J., Supple G.E. (2021). Periprocedural Acute Kidney Injury in Patients With Structural Heart Disease Undergoing Catheter Ablation of VT. JACC Clin. Electrophysiol..

[40] Deng F., Zhu C., Cao Y., Zhao S. (2025). Impact of Prolonged Elevated Heart Rate on Sepsis-Associated Acute Kidney Injury Patients: A Causal Inference and Prediction Study. Kidney Res. Clin. Pract..

[41] Hiraiwa H., Kasugai D., Okumura T., Murohara T. (2024). Clinical Implications of Septic Cardiomyopathy: A Narrative Review. Medicine.

[42] Dobson G.P., Letson H.L., Morris J.L. (2024). Revolution in Sepsis: A Symptoms-Based to a Systems-Based Approach?. J. Biomed. Sci..

[43] Evans L., Rhodes A., Alhazzani W., Antonelli M., Coopersmith C.M., French C., Machado F.R., Mcintyre L., Ostermann M., Prescott H.C. (2021). Surviving Sepsis Campaign: International Guidelines for Management of Sepsis and Septic Shock 2021. Intensive Care Med..

[44] Morelli A., Donati A., Ertmer C., Rehberg S., Kampmeier T., Orecchioni A., D’eGidio A., Cecchini V., Landoni G., Pietropaoli P. (2013). Microvascular Effects of Heart Rate Control with Esmolol in Patients with Septic Shock: A Pilot Study. Crit. Care Med..

[45] Tupper P., Redfern O., Harrison C.H., Gerry S., Biggs C., Walker B., Watkinson P. (2025). Is Age Associated with Different Vital Signs in Adults Presenting to Hospital with Bacterial Infection? A Systematic Review and Meta-Analysis. Age Ageing.

[46] Gudivada K.K. (2024). Targeted Heart Rate Control in Sepsis: A Promising Path or a Double-edged Sword?. Indian J. Crit. Care Med..

[47] Guarracino F., Ferro B., Morelli A., Bertini P., Baldassarri R., Pinsky M.R. (2014). Ventriculoarterial Decoupling in Human Septic Shock. Crit. Care.

[48] Ishmael L., Zalocha J. (2020). ST-Elevation Myocardial Infarction in the Presence of Septic Shock. Case Rep. Crit. Care.

[49] Efremidis M., Vlachos K., Kyriakopoulou M., Mililis P., Martin C.A., Bazoukis G., Dragasis S., Megarisiotou A., Unger P., Frontera A. (2021). The RV1-V3 Transition Ratio: A Novel Electrocardiographic Criterion for the Differentiation of Right versus Left Outflow Tract Premature Ventricular Complexes. Heart Rhythm O2.

[50] Liu W., Shao R., Zhang S., Jin L., Liu R., Chen P., Hu J., Ma H., Wu B., Liang W. (2024). Characteristics, Predictors and Outcomes of New-Onset QT Prolongation in Sepsis: A Multicenter Retrospective Study. Crit. Care.

[51] Kotruchin P., Chuehongthong M., Tangpaisarn T., Serewiwattana N., Phungoen P., Mitsungnern T., Buranasakda M. (2026). Association of Electrocardiogram Abnormalities with Clinical Outcomes in Emergency Department Sepsis Patients. West J. Emerg. Med..

