# Supplementary material for: Associations of Heart Rate Trajectories with Mortality and AKI Occurrence in Septic Patients: A Retrospective Study from MIMIC-IV and eICU Databases
Source: J Clin Med. 2026 Aug 28;15(17):6660. doi: 10.3390/jcm15176660 (PMC13566285; doi:10.3390/jcm15176660)
Supplement: Supplementary file 1 [file jcm-15-06660-s001.zip › figure S1-S4.pdf]

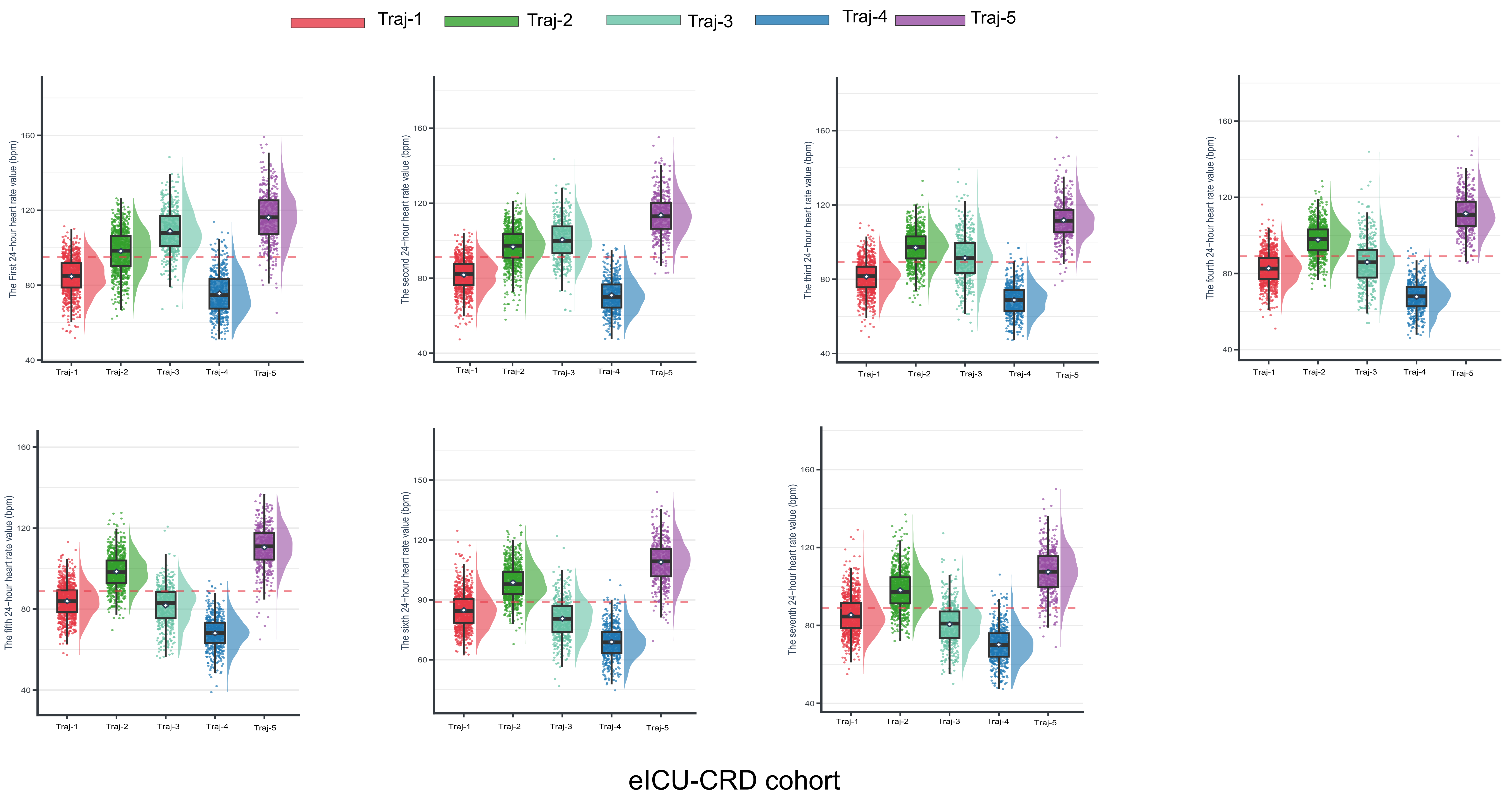

Figure S1 Dynamic changes in heart rate levels across five trajectories over 7 24-hour periods based on eICU-CRD cohort.

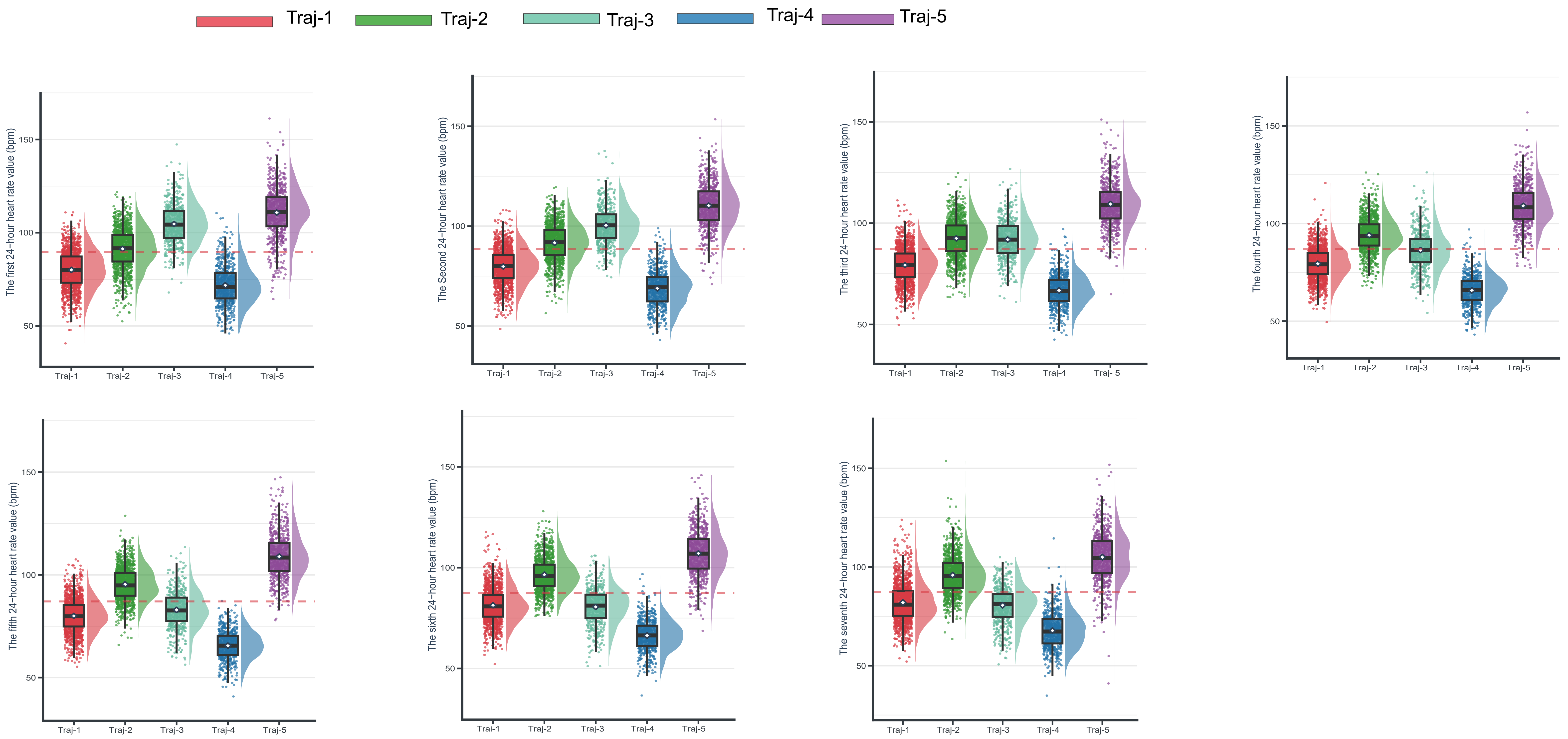

MIMIC-IV cohort

Figure S2 Dynamic changes in heart rate levels across five trajectories over 7 24-hour periods based on MIMIC-IV cohort.

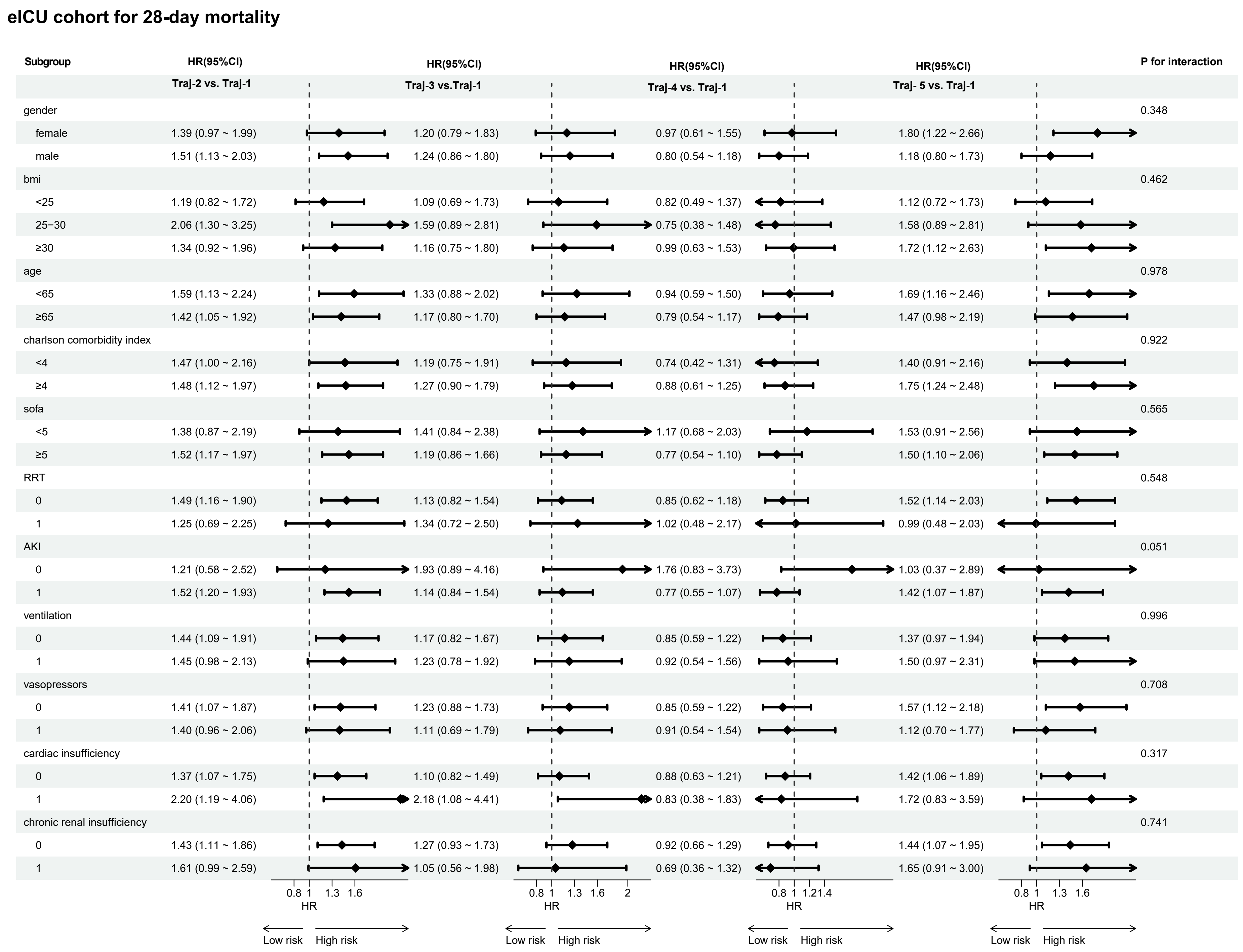

Figure S3. Forest plot of subgroup analyses for the association between heart rate trajectory groups and 28-day mortality based on eICU-CRD cohort.

MIMIC IV cohort for 28-day mortality

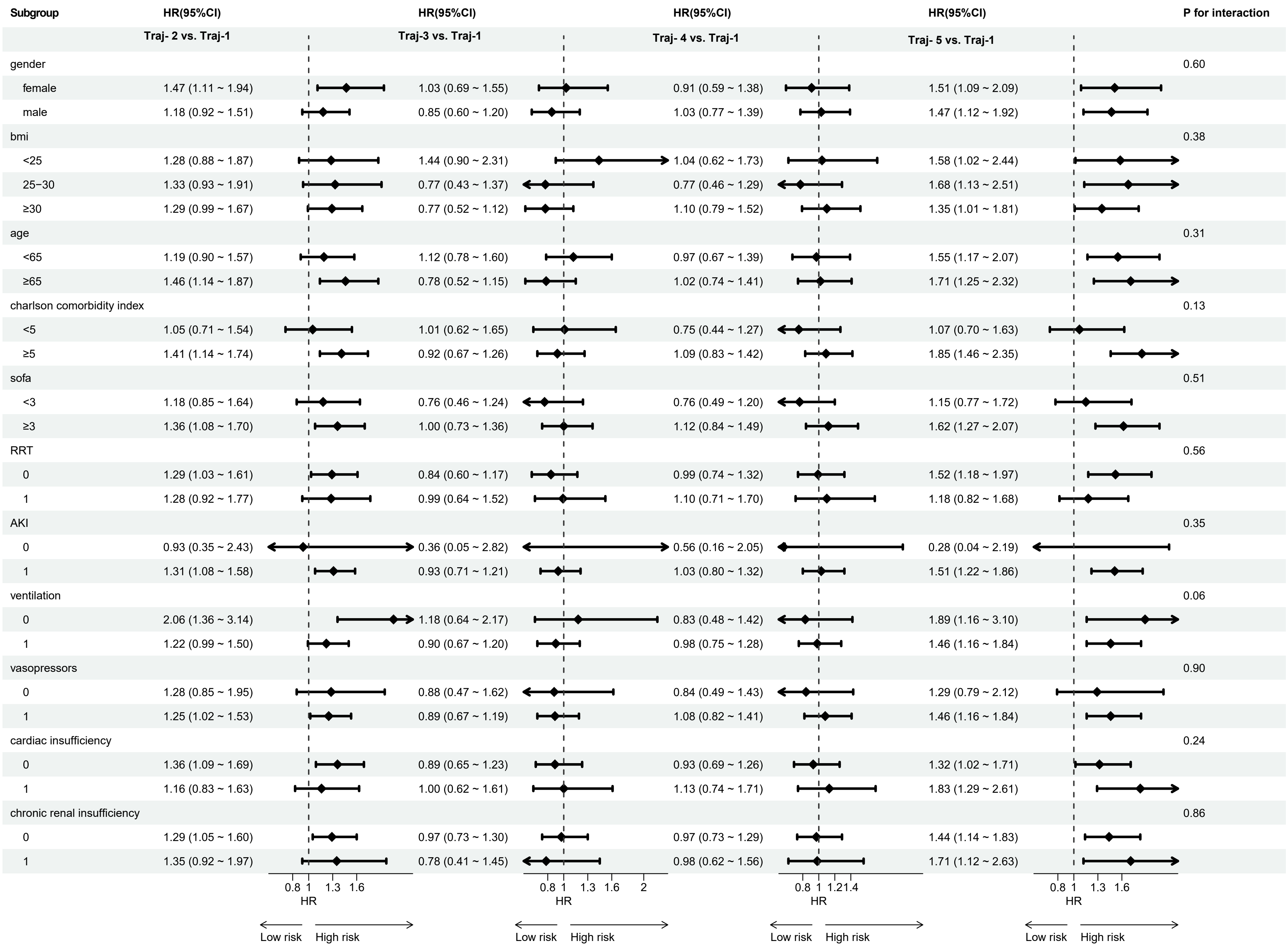

Figure S4. Forest plot of subgroup analyses for the association between heart rate trajectory groups and 28-day mortality based on MIMIC IV cohort.
